# Immunophenotypical and pathological changes in dogs experimentally infected with *Ehrlichia canis*

**DOI:** 10.1590/S1984-29612022020

**Published:** 2022-04-08

**Authors:** Márcio Botelho de Castro, Matias Pablo Juan Szabó, Lucia Padilha Cury Thomaz de Aquino, Ana Silvia Dagnoni, Antonio Carlos Alessi, Mirela Tinucci Costa, Andréa Cristina Higa Nakaghi, Mariele De Santi, Ana Claúdia Calchi, Marcos Rogério André, Rosangela Zacarias Machado

**Affiliations:** 1 Laboratório de Patologia Veterinária, Universidade de Brasília – UnB, Brasília, DF, Brasil; 2 Faculdade de Medicina Veterinária, Universidade Federal de Uberlândia – UFU, Uberlândia, MG, Brasil; 3 Departamento de Medicina Veterinária, Universidade do Estado de Santa Catarina – UDESC, Florianópolis, SC, Brasil; 4 Clínica Veterinária Vets Pets, Bady Bassitt, SP, Brasil; 5 Departamento de Patologia, Reprodução e Saúde Única, Faculdade Ciências Agrárias e Veterinárias – FCAV, Universidade Estadual Paulista – UNESP, Jaboticabal, SP, Brasil; 6 Departamento de Clínica e Cirurgia Veterinária, Faculdade Ciências Agrárias e Veterinárias – FCAV, Universidade Estadual Paulista – UNESP, Jaboticabal, SP, Brasil; 7 Universidade de Sorocaba – UNISO, Sorocaba, SP, Brasil

**Keywords:** Monocytic ehrlichiosis, canine, tick-borne disease, infection, immunity, Erliquiose monocítica, canino, doença veiculada por carrapatos, infecção, imunidade

## Abstract

Canine monocytic ehrlichiosis (CME) is one of the most important tick-borne diseases worldwide, with multisystemic presentations. Immune dysregulation has been proposed as the primary mechanism involved in its pathogenesis and in tissue injury in dogs with CME. Experimental infection of German Shepherd dogs in the present study demonstrated that CME caused marked pathological changes in their lymph nodes and spleen, and also gave rise to mononuclear infiltration in organs and tissues. Immunophenotyping of cells in lymph nodes, spleen and injured tissues highlighted differences in lymphocyte subsets, local expression of immunoglobulin subclasses and MHCII molecules between infected and control dogs. These findings suggest that the immunophenotypic and immunopathological changes in dogs with acute experimental CME are related to Th1 bias and compartmentalized immune response.

## Resumo

A erliquiose monocítica canina (EMC) é uma das doenças veiculadas por carrapatos com apresentações multisistêmicas mais relevantes em todo o mundo. A desregulação do sistema imune vem sendo proposta como o principal mecanismo envolvido na patogênese e lesão de tecidos em cães com EMC. A infecção experimental de pastores alemães nesta pesquisa evidenciou marcadas alterações patológicas em linfonodos, baço e também infiltração mononuclear em órgãos e tecidos. A imunofenotipagem de células em linfonodos, baço e tecidos lesados destacou diferenças em subconjuntos de linfócitos, expressão local de subclasses de imunoglobulinas e de moléculas MHCII entre cães infectados e controle. Esses achados sugerem que um viés Th1 e uma resposta imune compartimentalizada estão relacionados às alterações imunofenotípicas e imunopatológicas em cães com EMC experimental aguda.

## Introduction

Canine monocytic ehrlichiosis (CME) is an important infectious disease worldwide (Little, 2010). The etiological agent of CME is *Ehrlichia canis*, a tick-borne obligate intracellular bacterium transmitted by ticks of the *Rhipicephalus sanguineus* species group (Harrus & Waner, 2011).

CME is a multisystemic disease with acute, subclinical or chronic clinical presentations (Little, 2010). Its clinical signs and laboratory alterations depend on the stage of the disease, pathogenicity of different *E. canis* strains and presence of coinfections with other arthropod-borne pathogens, such as *Babesia canis vogeli* and *Hepatozoon canis* (Gal et al., 2007). Acute CME is characterized by high fever, anorexia, emaciation, hepatomegaly, splenomegaly, lymphadenopathy, cardiac and respiratory disturbance, and nervous and ocular changes (Little, 2010).

Pancytopenia, thrombocytopenia (Saito & Walker, 2016), hypergammaglobulinemia, and anemia are the main clinical pathological changes observed in infected dogs (Harrus & Waner, 2011). Gross lesions include generalized lymphadenopathy, splenomegaly, edema of the limbs, and disseminated petechial hemorrhage in organs, mucous membranes, and subcutaneous tissues. Microscopically, lymphoplasmacytic perivascular inflammatory infiltration is frequently reported in the nervous system, kidneys, lungs, liver and lymphoid tissues (Reardon & Pierce, 1981a; Castro et al., 2004; Harrus & Waner, 2011).

Immunological and redox imbalance or changes in lymphoid tissues such as hypergammaglobulinemia, disseminated tissue lymphoplasmacytic inflammatory infiltration, lymphoreticular hyperplasia, and increased MHC II expression are possibly involved in the pathogenesis of CME (Harrus et al., 1999; Castro et al., 2004; Silva et al., 2013; Harrus, 2015; Saito & Walker, 2016). Changes in lymphocyte subpopulations and effector cells and the cellular expression of IgM and IgG have been reported in the lymphoid organs of dogs with CME (Castro et al., 2004; Mylonakis et al., 2011).

Ehrlichial infections have been related to developing a Th1 response with IFN-γ secretion by CD4 T cells and changes to the CD4:CD8 ratio of T lymphocytes in peripheral blood, which may play both pathogenic and protective roles (Saito & Walker, 2016). Increases in the population of CD8+ cytotoxic T cells were detected in lymphoid organs and blood in experimental and natural cases of CME (Castro et al., 2004; Lorente et al., 2008). In contrast, differences in lymphocyte subsets in peripheral blood were not evidenced in dogs naturally infected by *E. canis* (Villaescusa et al., 2012). Spatial compartmentalization of the immune system in different organs and tissues leads to great diversity in responses to pathogens at a local level (Sathaliyawala et al., 2013; Quaresma, 2019) and may explain different observations in CME.

In most canine vector-borne infections, including CME, immune-mediated sequelae due to immune dysregulation related to the host immune response remain relatively poorly characterized (Day, 2011). Therefore, investigations on compartmentalized responses in target organs and tissues are desirable to elucidate the complex mechanisms involved in pathogen-host interactions. In this regard, our study evaluated immunopathological, and immunophenotypical changes in the lymph nodes and spleen of dogs with acute experimental CME, along with inflammatory tissue infiltrates in their organs.

## Materials and Methods

Ten healthy, male (5) and female (5), German Shepherd dogs aged 4–6 months that were seronegative for *E. canis* were randomly allocated into two groups. Five dogs formed the experimental group, and other five dogs were used as uninfected controls. The dogs in the experimental group were intravenously inoculated with 5 ml of whole blood from a dog infected with *Ehrlichia canis* Jaboticabal strain (Castro et al., 2004). Clinical examination and investigations to detect morulae in mononuclear cells through peripheral-blood Giemsa-stained smears from the ear vein were performed daily (Elias, 1992; Castro et al., 2004). Serum samples were tested for the specific IgG response to *E. canis* using a “dot-blot” ELISA kit (Immunocomb®, Biogal) prior to inoculation and 30 days post-inoculation (dpi), in order to evaluate serum conversion and the effectiveness of the experimental infection. All the dogs were kept in individual boxes and fed premium dog food and water ad libitum, and free of endo and ectoparasites during all the experimental protocols.

All the dogs were euthanized with prior sedation with xylazine followed by a lethal bolus injection with sodium thiopental according to the norms of the National Council for the Control of Animal Experimentation (Brasil, 2013) within 30 dpi during the acute phase of the disease. Necropsies were performed, and samples from the spleen, right prescapular lymph node, liver, lungs, kidneys, central nervous system (CNS), heart, pancreas, adrenals, and intestines were collected for histopathological evaluation. These samples were fixed in phosphate-buffered 10% formalin (pH 7.0) for 12 h, embedded in paraffin, and cut into 5-μm sections. These sections were mounted on slides, stained with hematoxylin and eosin, and analyzed under a light microscope.

Immunohistochemistry was performed on paraffin-embedded sections from lymph nodes, spleen, and organs with mononuclear inflammatory infiltration, obtained from both the infected and the control dogs. Macrophages and T and B lymphocyte subsets were immunolabeled using the indirect immunoperoxidase method (Table 1). Immunostained cells were counted in lymph nodes and spleen in both infected and control dogs, as previously reported (Castro et al., 2004). The frequencies of immunolabeled cells (%) in mononuclear inflammatory infiltrates in the affected organs (liver, kidneys, lungs, and CNS) were estimated in a total of 500 cells. Descriptive statistics were produced using GraphPad Prism software (GraphPad Software Inc., version 8.01). Immunolabeled cell subsets in lymph nodes and spleen were compared between the infected and control dogs using a T-test. This study was approved by the Ethics Committee on the Use of Animals, FCAV-UNESP, protocol number 2471/21.

**Table 1:** Antigen dilutions used in the immunostaining protocols, and sources of the primary antibodies.

Antigen	Antibody clone^*,◊,§,★^	Dilution	Source
CD79α	HM57	1:20	Dako Corp.
IgG1	Canine IgG1, polyclonal	1:3000	Bethyl Labs.
IgG2	Canine IgG2, polyclonal	1:2000	Bethyl Labs.
IgE	Canine IgE, polyclonal	1:600	Bethyl Labs.
CD3	Polyclonal	1:200	Dako Corp.
Myeloid/histiocyte	MAC387	1:200	Dako Corp.
CD68	PG-M1	1:50	Dako Corp.
MHCII	TAL.1B5	1:200	Dako Corp.

* Antigen retrieval: target retrieval solution 10x concentrate (Dako Corp.), 97 °C, 40 min, performed in a water bath;

◊ detection method: biotin-peroxidase-streptavidin (LSAB+ System, Dako Corp.);

§ chromogen: DAB = 3,3′-diaminobenzidine;

★ positive control: canine lymph node.

## Results

The infected dogs presented fever, pale mucosae, and lymph node enlargement between the 10^th^ and 14^th^ days post-infection (dpi). During this period, morulae were observed within monocytes in peripheral blood smears from all the animals of the infected group. Splenomegaly was clinically detected in two dogs with CME. All the inoculated dogs had developed antibodies against *E. canis* by the 30^th^ dpi, with extrapolated titers ranging from 1:80 to 1:320 (Immunocomb®, Biogal) (Castro et al., 2004).

### Gross findings

**Figure 1:**
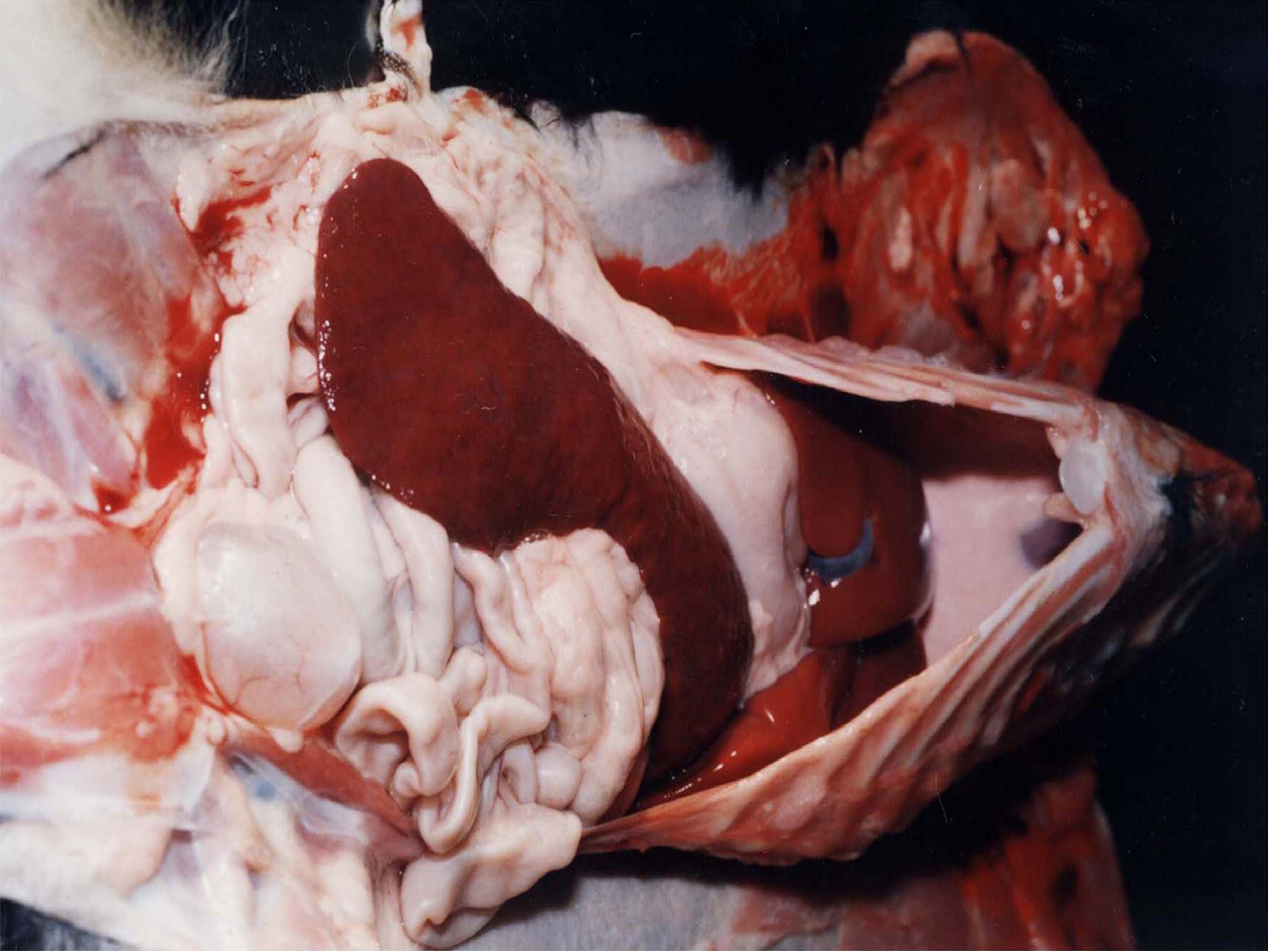
Dog with acute experimental CME. Marked splenomegaly.

The infected dogs showed marked anemia with paleness of mucous membranes and subcutaneous tissues, generalized lymphadenopathy, and splenomegaly (Figure 1). Enlarged lymph nodes were observed, with yellowish discoloration and petechiae in the medullary region. All the infected dogs had white-pulp hyperplasia and paleness of the liver and kidneys. Ascites (clear yellow liquid) and congested lungs were detected in two animals. No gross alterations were observed in the dogs of the control group.

### Histopathological findings

**Figure 2:**
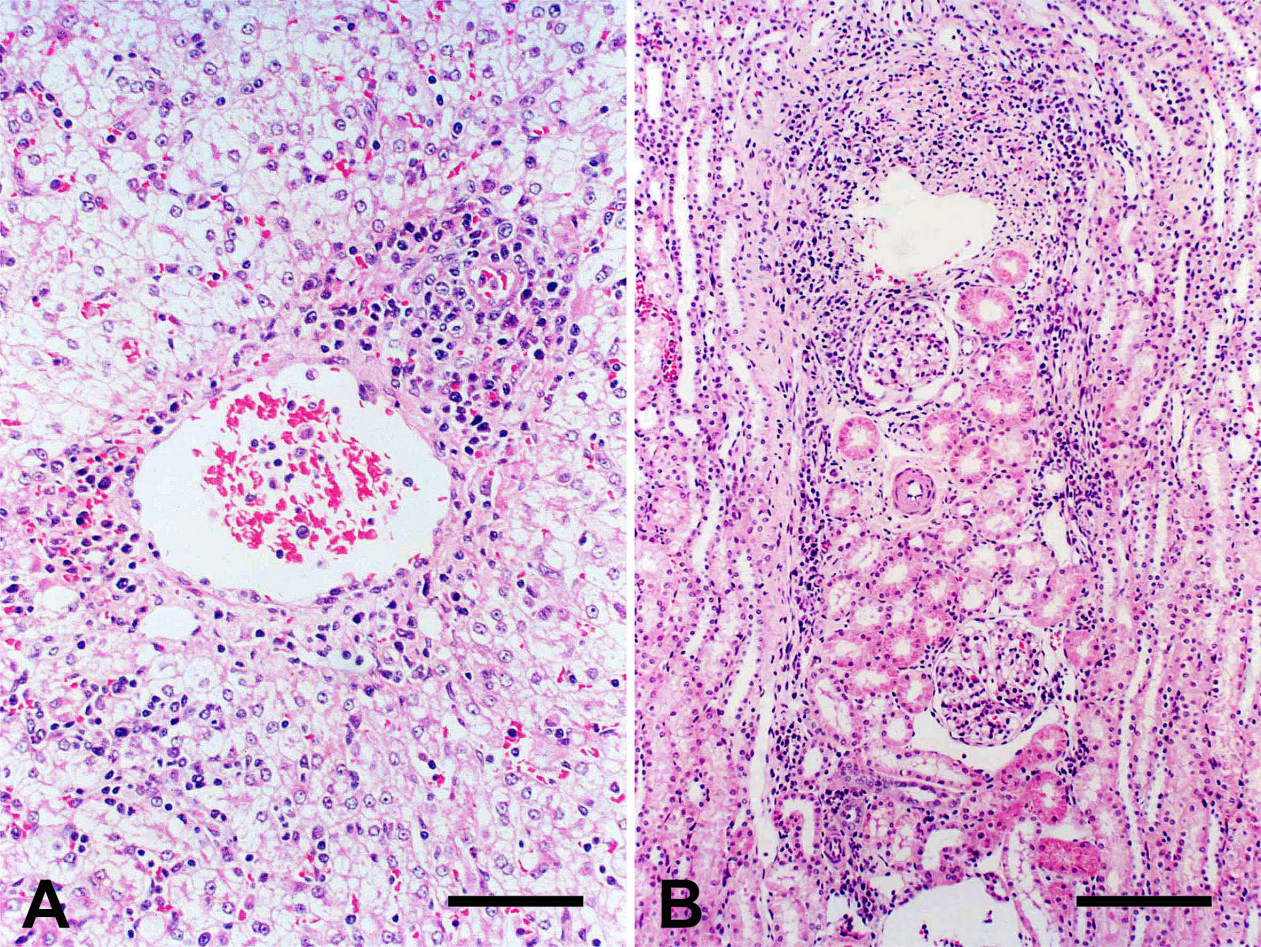
Dog with acute experimental CME. (A) Liver. Moderate hepatocyte swelling and perivascular mononuclear infiltrate in the portal region (H&E; bar = 50 µm); (B) Kidney. Mild interstitial and perivascular mononuclear infiltration (H&E, bar = 100 µm).

Microscopically, lymph nodes showed follicular hyperplasia within scattered tingible body macrophages, hyperplasia, marked plasmacytosis of medullary cords and sinusoidal histiocytosis. The spleen showed follicular hyperplasia with multifocal hemorrhages and congestion, plasmacytosis and hyperplasia of spleen cords, and sinus histiocytosis. Mild hydropic degeneration, mild to moderate mononuclear perivascular infiltrate, and sinusoid congestion were detected in the liver (Figure 2A). The kidneys showed mild to moderate diffuse interstitial mononuclear infiltrate and perivascular cuffing (Figure 2B). In the CNS, mild multifocal perivascular mononuclear cuffing and mild non-suppurative meningitis were also observed. The lungs showed mild lymphoplasmacytic perivascular infiltrate and mononuclear infiltrate that thickened the alveolar septa. The infected dogs also showed mild multifocal mononuclear perivascular infiltrate in the intestines and heart. No histological changes were detected in the other organs (heart, pancreas, adrenals, and intestines) of the infected dogs or control animals.

### Cellular immunophenotyping

The estimated densities of immunolabeled cells (cells x 10^2^/mm^2^) in lymph nodes and spleen are shown in Table 2. The percentages of immunolabeled cells in the inflammatory infiltrates in affected organs are shown in Table 3.

**Table 2:** Means and standard deviations (SD) of CD3, CD79α, IgG1, IgG2, IgE, MAC387, CD69 and MHC II labeled cell (cells × 10^2^/mm^2^) analyzed in different regions of the lymph nodes and spleen of infected and control dogs.

Organ	Region	Group	CD3	CD79α	IgG1	IgG2	IgE	MAC387	CD68	MHC II
Lymph node	Follicle	Control	1.4 ± 0.4	-	1.9 ± 1.5	4.1 ± 1.1	2.6 ± 0.5	0.3 ± 0.4	0.2 ± 0.1	23.7 ± 5.2
	Follicle	Infected	1.5 ± 0.3	-	1.2 ± 1.1	6.3 ± 2.9	1.2 ± 0.4*	0.1 ± 0.2	0.2 ± 0.1	15.7 ± 4.1*
	Paracortical	Control	-	9.5 ± 1.9	0.1 ± 0.1	2.5 ± 1.0	12.0 ± 4.7	0.6 ± 0.5	0.9 ± 0.6	17.1 ± 2.7
	Paracortical	Infected	-	10.4 ± 2.9	0.3 ± 0.0	2.3 ± 0.5	2.1 ± 2.5*	0.9 ± 0.8	0.4 ± 0.3	15.6 ± 1.4
	Medulla	Control	22.2 ± 2.9	23.2 ± 4.6	11.9 ± 8.4	20.0 ± 6.2	25.8 ± 6.2	2.7 ± 3.2	9.0 ± 4.6	24.7 ± 3.6
	Medulla	Infected	30.7 ± 7.7*	38.3 ± 4.9*	4.3 ± 2.4	35.3 ± 5.1*	10.2 ± 5.6*	2.7 ± 2.8	9.1 ± 2.8	15.6 ± 4.4*
Spleen	Follicle	Control	6.3 ± 1.2	-	0.9 ± 0.8	2.1 ± 0.8	7.6 ± 2.2	0.2 ± 0.4	0.5 ± 0.3	26.8 ± 6.2
	Follicle	Infected	6.4 ± 2.2	-	0.3 ± 0.5	2.9 ± 1.4	8.0 ± 3.8	0.1 ± 0.1	0.9 ± 1.2	14.7 ± 2.0*
	Marginal zone	Control	28.8 ± 5.1	44.4 ± 1.4	1.1 ± 1.2	9.0 ± 2.0	47.3 ± 4.4	0.0 ± 0.0	7.5 ± 1.5	30.1 ± 5.1
	Marginal zone	Infected	37.2 ± 2.6*	33.1 ± 3.3*	0.8 ± 0.5	37.8 ± 10.2*	33.4 ± 5.3*	0.6 ± 0.8	9.2 ± 2.6	16.2 ± 1.8*
	Splenic cords	Control	18.6 ± 6.2	10.4 ± 1.9	0.5 ± 0.3	3.0 ± 1.5	9.6 ± 3.1	13.6 ± 4.4	9.1 ± 2.3	19.6 ± 3.0
	Splenic cords	Infected	31.6 ± 3.1	15.0 ± 3.1*	0.8 ± 0.5	13.4 ± 4.2*	3.7 ± 0.6*	18.5 ± 4.6	12.6 ± 1.3*	10.8 ± 1.3

* p<0.05: differences between groups in the same region analyzed and antibody; - not determined.

**Table 3:** Immunophenotyping of cells within perivascular inflammatory infiltrates in organs of dogs experimentally infected with *Ehrlichia canis*.

Antigen	Organ (%)			
	Liver	Kidneys	Lungs	CNS
CD3	58.4 ± 9.2	67.8 ± 3.6	39.2 ± 3.3	80.6 ± 1.8
CD79α	28.8 ± 2.6	31.8 ± 4.5	27.0 ± 2.9	10.2 ± 1.5
IgG1	1.1 ± 0.5	2.2 ± 0.8	0.7 ± 0.3	1.1 ± 1.1
IgG2	31.0 ± 3.1	32.0 ± 7.7	27.0 ± 8.2	11.0 ± 2.6
IgE	3.0 ± 1.2	0.8 ± 0.8	0.5 ± 0.3	0.0 ± 0.0
MAC387	3.6 ± 2.5	0.8 ± 0.3	1.0 ± 0.0	0.0 ± 0.0
CD68	0.7 ± 0.8	0.7 ± 0.4	0.0 ± 0.0	0.0 ± 0.0
MHCII	26.0 ± 3.8	20.2 ± 1.5	33.6 ± 4.7	-

- absence of immunolabeled cells.

**Figure 3:**
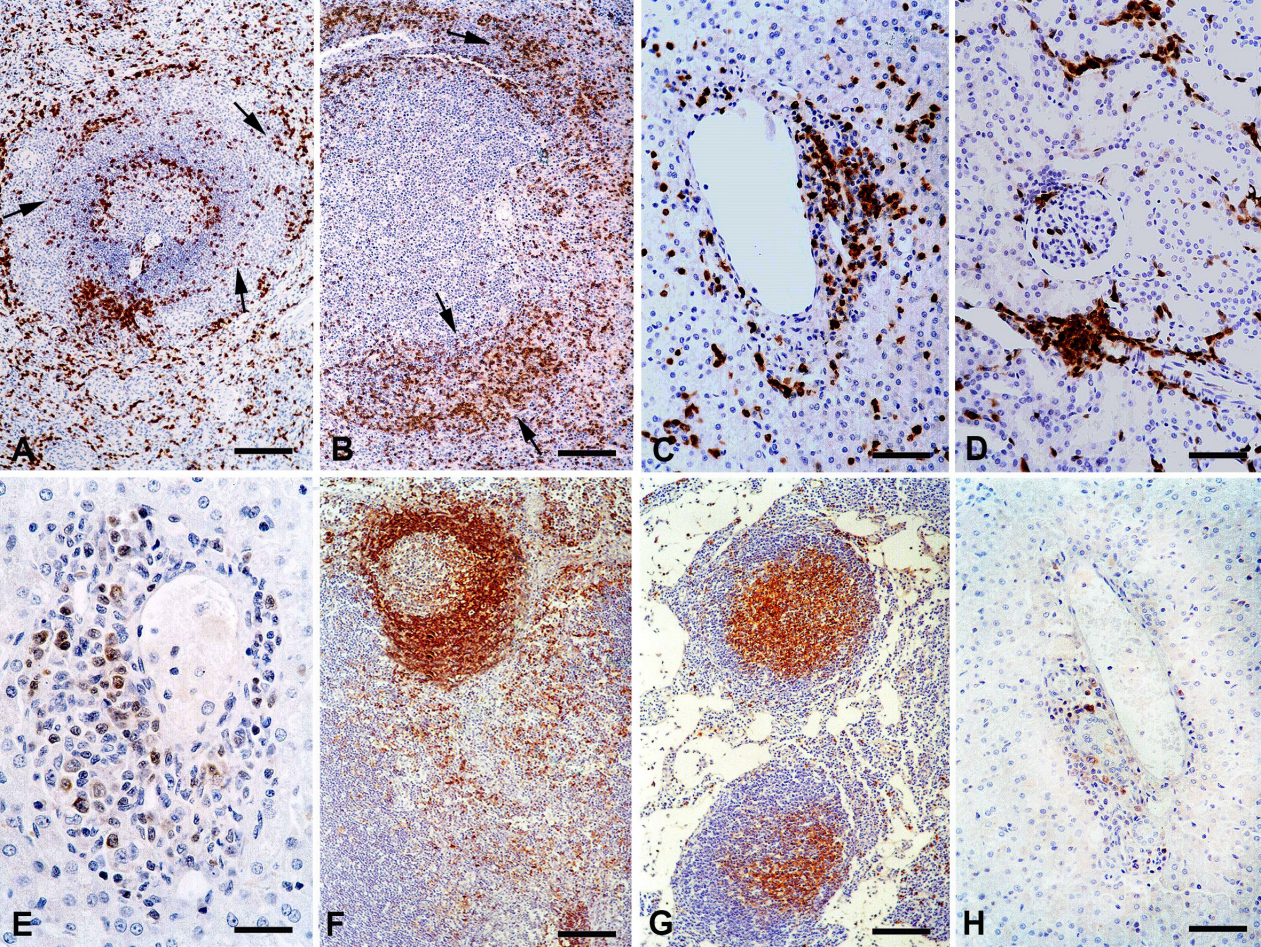
(A) Control dog, spleen. Marginal zone with mild immunostaining of mononuclear cells (arrows) (CD3 antibody, immunoperoxidase; bar = 100 µm); (B) Infected dog, spleen. Increased immunostaining of mononuclear cells (arrow) within the marginal zone (CD3 antibody, immunoperoxidase; bar = 100 µm); (C) Infected dog, liver. Perivascular inflammatory infiltrate with numerous immunolabeled T cells (CD3 antibody, immunoperoxidase; bar = 50 µm); (D) Infected dog, kidney. Interstitial and perivascular inflammatory infiltrates with numerous immunostained T lymphocytes (CD3 antibody, immunoperoxidase; bar = 50 µm); (E) Infected dog, liver. A few immunolabeled B lymphocytes within the perivascular inflammatory infiltrate (CD79α antibody, immunoperoxidase; bar = 25 µm); (F) Control dog, lymph node. Follicles and paracortical region with strong immunostaining (IgE antibody, immunoperoxidase; bar = 100 µm); (G) Infected dog, lymph node. Reduced follicular immunostaining and rare immunolabeled cells within the paracortical area (arrow) (IgE antibody, immunoperoxidase; bar = 100 µm); (H) Infected dog, liver. Rare immunolabeled macrophages within the perivascular inflammatory infiltrate (CD68 antibody, immunoperoxidase; bar = 100 µm).

CD3+ T lymphocyte levels were increased in the medullary region of the lymph nodes, splenic marginal zones (Figure 3A and 3B) and red pulp of the infected dogs (p < 0.05), in comparison with the control dogs. No CD3+ cell counts could be conducted in the paracortical region of the lymph nodes due to massive immunostaining of an anatomical area that was densely populated with T cells. CD3+ T cells were the most frequent lymphocyte subset within inflammatory infiltrates (p < 0.05) in the organs analyzed (Figure 3C and 3D).

CD79α+ B lymphocyte counts (p < 0.05) in the medullary region of lymph nodes and splenic white pulp were higher in the infected dogs than in the control animals (Table 2). The splenic marginal zone of the infected dogs showed a lower number of CD79α+ cells (p < 0.05) than in the control group. No CD3+ cell counts could be conducted in the paracortical region of the lymph nodes due to massive immunostaining since a dense T cell population typically occupies this anatomical area. No cell counts could be conducted in follicles due to the diffuse immunostaining of CD79α+ cells in the lymph nodes and spleen in both groups. CD79α+ B lymphocytes represented a smaller subset of cells in the inflammatory infiltrates (Figure 3E) in all affected organs (p < 0.05) than did CD3+ T lymphocytes.

The number of IgG1-expressing cells in the lymphoid organs did not differ between the infected and control groups. Inflammatory infiltrates in organs also showed a low number of IgG1+ cells. In contrast, more cells expressing IgG2 were detected in the medullary region of lymph nodes and in the marginal zone and splenic cords of infected dogs (p < 0.05), compared with control animals. Inflammatory infiltrates in organs had similar frequencies of IgG2+ cells and CD79α+ B lymphocytes (p > 0.05). The infected dogs had low proportions of IgE-expressing cells in all regions of lymph nodes (Figure 3F and 3G), and in the marginal zone and spleen cords (p < 0.05), compared with the control group. IgE+ cells presented low frequency in inflammatory infiltrates in most organs.

MAC387+ and CD68+ cells had similar counts in the infected and non-infected dogs (p > 0.05) in most lymph nodes and spleen regions. CD68+ macrophage levels were higher in the splenic cords of infected dogs than in the control group (p < 0.05). Immunophenotyping showed low proportions of MAC387+ and CD68+ cells (Figure 3H) within inflammatory infiltrates in the liver, kidneys, lungs and CNS.

**Figure 4:**
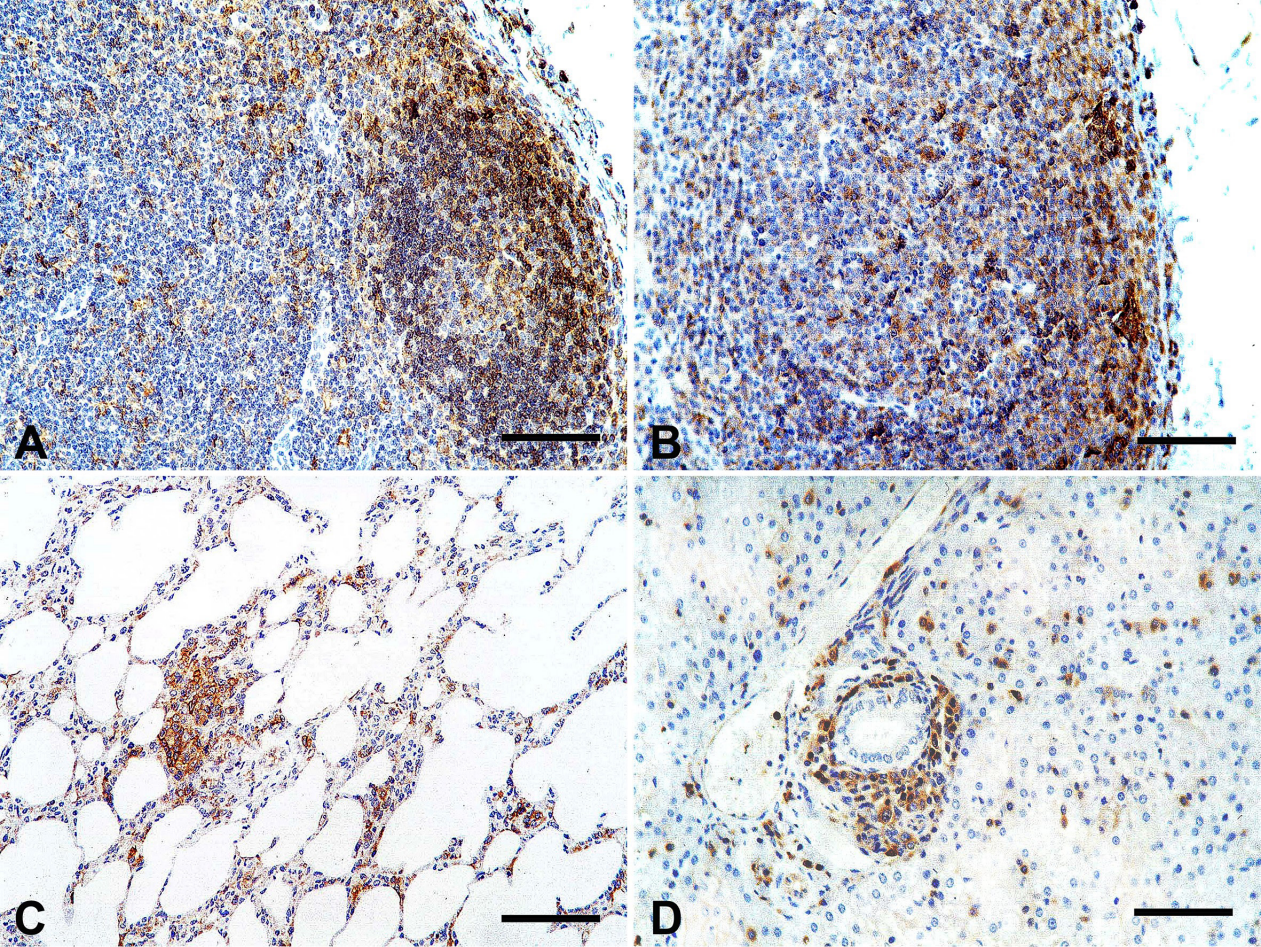
(A) Control dog, lymph node. Sparse cells immunolabeled in the follicular region (germinal center) (MHCII antibody, immunoperoxidase; bar = 50 µm); (B) Infected dog, lymph node. Increased mononuclear immunolabeled cells within the germinal center (MHCII antibody, immunoperoxidase; bar = 100 µm); (C) Infected dog, lung. Mononuclear cells with strong immunostaining in the perivascular and interstitial inflammatory infiltrates (MHCII antibody, immunoperoxidase; bar = 100 µm); (D) Infected dog, lung. Immunostaining of mononuclear cells in the perivascular cuffing (MHCII antibody, immunoperoxidase; bar = 100 µm).

The infected dogs showed lower numbers of cells expressing MHC class II molecules in most regions analyzed in the lymphoid organs (Figure 4A and 4B), compared with the control group (p < 0.05). There were no differences in MHCII molecule expression in the paracortical region of lymph nodes between the groups (p > 0.05). MHCII+ cells had notable frequency (18.7% to 38.3%) within the inflammatory infiltrates in most organs (Figure 4C and 4D) of the infected dogs.

## Discussion

Canine monocytic ehrlichiosis (CME) is one of the most important tick-borne diseases of dogs worldwide. The pathogenesis of CME remains unclear, especially regarding comprehension of the immunopathological changes in lymphoid organs and the development of tissue injuries. Previous studies have already revealed the vast complexity of host immune responses against *Ehrlichia* and the variety of evasion mechanisms of rickettsial organisms (Winslow et al., 2003; Winslow & Bitsaktsis, 2005). In addition, immune system imbalance has been proposed as a major mechanism for CME pathogenesis (Harrus et al., 2001b; Harrus et al., 2003; Castro et al., 2004).

All the dogs in the infected group of our study developed fever and demonstrated morulae within the cytoplasm of mononuclear cells and clinical signs, between ten and fourteen days post-infection, as previously reported for CME (Castro et al., 2004; Harrus et al., 2012). Variations in the onset of clinical signs can be related to the parasite strain (Perez et al., 1996; McBride et al., 2003) or to the high susceptibility to CME of some dog breeds such as German Shepherd dogs (Nyindo et al., 1980). Seroconversion against *E. canis* (IgG) was demonstrated 30 days post-infection in all the infected dogs. Dogs with CME develop specific IgM and IgG production against *E. canis* between seven and 21 days post-infection (McBride et al., 2003; Harrus et al., 2012), as observed in the experimentally infected group of our study.

Pallid mucous membranes and subcutaneous tissues, lymphadenopathy, and splenomegaly were the main clinical signs detected in the dogs with acute experimental CME, as previously reported (Oliveira et al., 2000; Castro et al., 2004; Harrus et al., 2012). The gross findings among the dogs of the infected group consisted mainly of anemia, moderate lymphadenopathy, marked splenomegaly with red pulp hyperplasia, and pulmonary congestion. These findings have been reported both in natural and in experimental CME and are related to marked immune stimulation during the infection (Keefe et al., 1982; Reardon & Pierce, 1981a, b; Harrus et al., 2012). The ascites detected in two infected dogs in the present study may have been related to hypoalbuminemia and polyclonal gammopathy, which have been observed in some cases of CME (Harrus et al., 1996).

The histopathological changes observed in all infected dogs mostly reflected the increases in cellularity and size of the lymph nodes, spleen, and perivascular mononuclear inflammatory infiltration in most organs. The marked plasmacytosis and hyperplasia of medullary cords in lymph nodes and splenic cords possibly evidenced exacerbated immune humoral stimulation and differentiation of B lymphocytes to effector cells (plasma cells) in dogs with acute CME. The detection of anti-*E. canis* IgG antibodies in all the dogs at 30 days post-infection sustained this hypothesis. Additionally, the mild increase in T lymphocyte areas in the spleen and lymph nodes also demonstrated a concomitant cell-mediated immune response in dogs with CME. The pathological findings detected in the lymphoid organs of the infected dogs in this experiment were similar to those previously reported (Reardon & Pierce, 1981a; Castro et al., 2004). Experimental immunosuppression applied to dogs through use of glucocorticoids demonstrated improvement in the clinical course of CME and regression of pathological changes in lymphoid organs, which suggested that participation of the immune system in the development of lymphoid tissue lesions was important (Reardon & Pierce, 1981a).

In addition to the morphological changes detected in the lymph nodes and spleen, variable degrees of multifocal perivascular mononuclear inflammatory infiltration in most organs, such as the liver, kidneys, lungs, central nervous system (CNS), heart, and intestines, were the primary lesions and pathological hallmarks of the acute experimental CME in the German Shepherd dogs of the present study. Similar sets of pathological findings had previously been reported in experimental and natural infections by *E. canis* in dogs (Hildebrandt et al., 1973; Reardon & Pierce, 1981a; Castro et al., 2004). *Ehrlichia* antigens have been detected free in the plasma of dogs with CME and mice experimentally infected with *E. chaffeensis* (Waner et al., 1996; Li & Winslow, 2003). Additionally, infected cells in the peripheral circulation may adhere to the endothelium, thus inducing a vascular inflammatory process and tissue damage (Lepidi et al., 2000). Given the widespread distribution of perivascular inflammation in organs and tissues, it is possible to hypothesize that vascular deposition of free plasma ehrlichial antigens or infected mononuclear cells adhering to the endothelium in dogs with CME may trigger vascular tissue damage and inflammation. In CME, the deposition of circulating immune complexes has been suggested as the cause of damage in the vascular system (Harrus et al., 2001a).

Immunophenotypical changes to mononuclear cell populations were detected in the lymph nodes and spleen of the infected dogs of the present study, compared with the control group. An increased population of CD3+ T lymphocytes was evidenced in the marginal zones of follicles and the medulla of lymph nodes, and in the splenic white pulp. Additionally, massive plasmacytosis and increased CD79α+ B lymphocyte populations in the medullary region of lymph nodes and splenic cords were evidenced in infected dogs, compared with the control group. Similarly, immunophenotypical changes in T and B lymphocyte subpopulations and increased CD8+ T cells in lymph nodes and spleen were previously observed in German Shepherd dogs with experimental CME and increased immune cellular humoral activity was demonstrated (Castro et al., 2004). In contrast, no differences in CD3+ and CD79α+ lymphocyte subpopulations were detected in the peripheral blood of dogs with clinical and subclinical CME (Villaescusa et al., 2012). The variable pathogenic potential of *E. canis* strains (Aguiar & Melo, 2015; Nambooppha et al., 2018), along with the compartmentalization of immune responses to some infectious agents (Quaresma, 2019), may explain the differences in lymphocyte subpopulations between lymphoid organs and peripheral blood in dogs with CME.

CD3+ T lymphocytes were the predominant subset of mononuclear cells within perivascular inflammatory infiltrates in injured organs and tissues, followed by CD79α+ B lymphocytes in the infected dogs of the present study. In a murine model of ehrlichial infection, cytotoxic T cell and natural killer T cell (NKT) activation in a specific profile of cytokine overproduction in a Th-1 immune response was correlated with exacerbated tissue damage and fatal outcomes (Dierberg & Dumler, 2006; Tominello et al., 2019).

One of the most important immunopathological changes detected in the dogs with CME of the present study was the increase of IgG2+ cells and the decrease of IgG1+ and IgE+ cells, which expressed immunoglobulins in the membrane or inside the cytoplasm in the lymph nodes, spleen, and perivascular inflammatory infiltration in organs and tissues. Furthermore, IgG2+ cells and CD79α+ B lymphocytes were similar in numbers in the lymph nodes, spleen, and perivascular inflammatory infiltrates. A high plasmatic IgG2 subclass response was previously detected in cases of natural and experimental infection by *E. canis* at different phases of the disease, in both asymptomatic and symptomatic dogs (Harrus et al., 2001b). Marked IgG expression by mononuclear cells within lymph nodes and spleen was detected in acute experimental infection of German Shepherd dogs (Castro et al., 2004). Therefore, here, we highlight the importance of IgG2 production in response to the infection observed *in situ* for the first time in lymphoid organs and inflammatory infiltration in several organs of dogs with acute CME.

Regarding the *in situ* production of immunoglobulin subsets in dogs with acute CME, the profile characterized by high cellular expression of IgG2 and reduction or low numbers of IgG1+ and IgE+ cells supports the notion that polarization towards a Th1-type immune response occurs (Harrus et al., 2001b; Tajima & Rikihisa, 2005) in the lymph nodes, spleen and perivascular inflammatory infiltrates in affected organs and tissues. A Th1-type immune response characterized by IFN-γ and IgG2 antibody production was found to be predominant in dogs with mild infection by the *E. canis* Oklahoma strain (Tajima & Rikihisa, 2005). This was also suggested by the immunophenotype of mononuclear cells in dogs with subclinical ehrlichiosis (Lorente et al., 2008), and in a murine fatal experimental model and in humans with monocytotropic ehrlichiosis (Ismail et al., 2004; Walker, 2005). Our observations are underscored by the fact that the acute infection of dogs with *E. canis* Jaboticabal strain gave rise to increased expression of TNF-α and peaks of IFN-γ, which are hallmarks of a Th1 profile of cytokines production (Faria et al., 2011; McBride & Walker, 2011; Mansueto et al., 2012).

The mechanisms for infection-induced tissue injury have not been entirely elucidated in CME (Tajima & Rikihisa, 2005; Saito & Walker, 2016). In some rickettsial infections, the Th1 profile of cytokine expression is determinantal in the pathogenesis and disseminated vascular damage and inflammatory events (Stevenson et al., 2008; McBride & Walker, 2011). Exacerbated production of reactive oxygen species (ROS) such as hydrogen peroxide, superoxide anion, and hydroxyl radicals, induced by a combination of IFN-γ and TNF-α activity, has been proposed as a possible mechanism for tissue injury (Day, 2011; Mansueto et al., 2012). A state of redox imbalance characterized by high serum levels of nitrite/nitrate, lipid peroxidation products, advanced oxidation protein products, and glutathione reductase activity was observed in dogs experimentally infected with the *E. canis* Jaboticabal strain (Silva et al., 2013). Our observations on the *in situ* immunoglobulin profile production in dogs with CME support the notion that a Th1 immune response plays a role in tissue injury and changes in the mononuclear cell subsets within lymphoid organs such as the lymph nodes and spleen.

No significant changes in populations of MAC387+ and CD68+ macrophages were detected in the dogs with acute experimental CME in the present study, except a mild increase in CD68+ cells within splenic cords. Additionally, rare macrophages were immunostained in inflammatory infiltrates in affected organs. Histiocytosis has been quite often reported in lymphoid organs, and inflammation has been reported in the injured organs and tissues of dogs with chronic CME (Hildebrandt et al., 1973; Harrus et al., 2012) and may vary in intensity in the course of infection (Reardon & Pierce, 1981a). Considering that IFN-γ triggers antimicrobial mechanisms in macrophages that have a central role in the elimination of *Ehrlichia* (Mansueto et al., 2012), non-numerical variation in subpopulations of macrophages in lymphoid organs and low frequency of macrophages in injured tissues of dogs with acute experimental CME may be related to some ehrlichial mechanism for immune evasion. Interference in signal transduction may prevent activation of the bactericidal properties of leukocytes, and this has been proposed as another ehrlichial evasion mechanism (Rikihisa, 2000).

The reduction in MHCII expression in the lymph nodes and spleen cells that we observed in the dogs with acute experimental CME may have indicated downregulation during the infection. Similar to our findings, a reduction in MHCII expression in DH82 cells (dog macrophages/histiocytes) infected with *E. canis* was previously demonstrated, and it was suggested as a possible pathway for *E. canis* to escape from the immune system (Harrus et al., 2003). A reduction in MHCII expression may explain the persistence of ehrlichial infection in the spleen of dogs for several years (Harrus et al., 1998). The expression of class II major histocompatibility complex molecules was found to be determinantal for activating macrophages and eliminating ehrlichial organisms in experimental infection of mice by *E. chaffeensis* (Ganta et al., 2002, 2004). Downregulation of class II antigen expression in response to IFN-γ has been proposed as a strategy for ehrlichial survival in the face of the microbicidal leukocytes’ activity (Rikihisa, 2000).

In contrast to the MHCII expression in mononuclear cell populations in lymphoid organs, MHCII+ cells represented 20-30% of the inflammatory cells in the injured tissues of the infected animals of the present study. Additionally, the low number of macrophages and the frequencies of MHCII+ cells in those locations were quite similar to the frequency of CD79α+ cells, which suggested that B lymphocytes were responsible for the expression of MHCII molecules. The increased local expression of MHCII and the predominance of the CD3+ T lymphocytes subset within the inflammatory infiltrate in the dogs with acute CME suggested that an immune-mediated mechanism of injury has led to perivascular inflammation in the affected tissues in response to the infection. Ehrlichial infections may provide Th1 differentiation (Ismail et al., 2004; McBride & Walker, 2011) with excessive cytotoxic T cell and macrophage activation, thereby generating tissue-damaging effector cells (Dierberg & Dumler, 2006). Additionally, high TNF-α expression previously detected in dogs infected by the same *E*. *canis* Jaboticabal strain (Faria et al., 2011) possibly produced a similar local pathogenic immune imbalance in the experimental group in our study. Furthermore, NKT cells may promote apoptosis of macrophages and upregulation of antigen-presenting cell activity, thus contributing to the induction of pathogenic T-cell responses (Tominello et al., 2019).

## Conclusions

Herein, we have provided the first description of immunophenotyping of inflammatory infiltrates in injured tissues of dogs with acute CME, essential information towards elucidating the pathogenesis of *E*. *canis* infections. The set of immunophenotypical changes in these dogs with acute experimental CME suggests a Th1 bias in both lymphoid tissues and within perivascular inflammation in organs. Differences in the expression of MHCII molecules between lymphoid tissues and inflammatory infiltrates in organs may reflect the compartmentalization and modulation of the immune response in infected dogs. These findings have highlighted the *in situ* changes in the subsets of mononuclear cells in affected tissues during *E*. *canis* acute infections, which may present variations within different immunologic compartments.
